# Genome-Wide Association Studies In Plant Pathosystems: Toward an Ecological Genomics Approach

**DOI:** 10.3389/fpls.2017.00763

**Published:** 2017-05-23

**Authors:** Claudia Bartoli, Fabrice Roux

**Affiliations:** LIPM, Centre National de la Recherche Scientifique, Institut National de la Recherche Agronomique, Université de ToulouseCastanet-Tolosan, France

**Keywords:** genome-wide association mapping, disease resistance, pathogenicity, crops, microbiota, pathobiota, genome-to-genome analysis, co-evolution

## Abstract

The emergence and re-emergence of plant pathogenic microorganisms are processes that imply perturbations in both host and pathogen ecological niches. Global change is largely assumed to drive the emergence of new etiological agents by altering the equilibrium of the ecological habitats which in turn places hosts more in contact with pathogen reservoirs. In this context, the number of epidemics is expected to increase dramatically in the next coming decades both in wild and crop plants. Under these considerations, the identification of the genetic variants underlying natural variation of resistance is a pre-requisite to estimate the adaptive potential of wild plant populations and to develop new breeding resistant cultivars. On the other hand, the prediction of pathogen's genetic determinants underlying disease emergence can help to identify plant resistance alleles. In the genomic era, whole genome sequencing combined with the development of statistical methods led to the emergence of Genome Wide Association (GWA) mapping, a powerful tool for detecting genomic regions associated with natural variation of disease resistance in both wild and cultivated plants. However, GWA mapping has been less employed for the detection of genetic variants associated with pathogenicity in microbes. Here, we reviewed GWA studies performed either in plants or in pathogenic microorganisms (bacteria, fungi and oomycetes). In addition, we highlighted the benefits and caveats of the emerging joint GWA mapping approach that allows for the simultaneous identification of genes interacting between genomes of both partners. Finally, based on co-evolutionary processes in wild populations, we highlighted a phenotyping-free joint GWA mapping approach as a promising tool for describing the molecular landscape underlying plant - microbe interactions.

## Introduction

In the last decade the World Health Organization (WHO) reported more than 300 newly infectious diseases that have emerged as threat for human (Jones et al., 2008). In the same trend, a conspicuous burst of plant diseases has been reported since the beginning of this century (Bartoli et al., 2016). Newly pathogenic microbes can spill over form reservoirs or newly variants of a pre-existing pathogen (Elena et al., 2011). Three main driving factors for Emerging Diseases (ED) occurrences are currently considered in both human and plant pathology: (i) genetic and biological factors both acting on the host and the etiological agent (i.e., changes in host susceptibility and microbial adaptation *via* genomic rearrangements), (ii) ecological and environmental factors (i.e., climate change, perturbations of the ecological niches of the pathogens and changes in host demography), (iii) social, political and economic factors (i.e., land use and international travel; Smolinski et al., 2003). Whatever the factor we consider the common line in ED is the evolutionary potential of microbes that through genetic changes can bypass the host defense system by spreading over new host populations. However, the evolutionary genetic change of the pathogen is not sufficient for the occurrence of ED and ecological perturbations such as habitat modifications are necessary for the newly pathogen to encounter its host. For example, agricultural practices can drastically alter the environment by offering routes for transmission of pathogens (Institute of Medicine, 2003).

As proposed by (Engering et al., 2013), ED caused by microbial agents can be divided in three major groups. The first disease emergence category concerns those pathogens that emerge in a novel host, a common process that is also called host jump. Pathogenic lines with flexible genomes (or high evolvability behavior) and with high environmental survival are much more prone for host jumps (Engering et al., 2013), although interspecies contact rate—that is burst by the ongoing growth in human population and consumption of animal products—is one of the main drivers for host jump occurrence. For example, the oomycete species *Phytophthora infestans* responsible for the Irish potato famine, experienced several host jumps because of the high rate of genomic rearrangements in non-coding genomic regions (Raffaele et al., 2010). A second type of disease emergence involves mutant pathogenic lines that caused more severe diseases after the acquisition of novel genetic traits. Novel pathogenicity traits can be the result of mutations or horizontal gene transfer and both processes increase pathogen's genetic variability (Bartoli et al., 2016). In light of this, the barley powdery mildew, *Blumeria graminis*, has increased its pathogenicity and has acquired an obligate biotrophy life-style *via* retrotransposon proliferation and gene loss (Spanu et al., 2010). However, the environment where the pathogenic populations evolve is of fundamental importance for triggering the evolution of new traits increasing aggressiveness in a pathogen. For example, the intensification of antibiotic utilization in agriculture increased antibiotic resistance gene acquisition as well as virulence in several bacterial pathogens (Jones et al., 2008). Examples are the *Escherichia coli* O157:H7 in which its aggressiveness increased after the acquisition of the Shiga toxin plasmid, or the rice fungal pathogen *Cochiliobolus miyabeanus* responsible of Great Bengal Famine of 1943 that gaining non-host specific toxins raised virulence on the rice host (Bruyne et al., 2016). However, in some cases, resistance to antibiotics can also naturally occur in pathogens without the selective pressure of the molecule. For example, in *Pseudomonas viridiflava* hypermutable variants, resistant to several antibiotics, spontaneously occur in synthetic media, as well *in planta*, in absence of antibiotics (Bartoli et al., 2015). Also, mutations CYP51 conferring azole resistance in different fungal pathogens is not always related to the utilization of fungicides as demonstrated in *Fusarium* spp (Parker et al., 2014). Lastly, the geographic expansion (or geographic jump) of a pathogen is also a form of disease emergence that can rapidly lead to disease epidemics (Engering et al., 2013). As already mentioned for host jumps, pathogens with more flexible genomes are obviously more prone to expand their geographic range because they can rapidly respond to the environmental conditions of a new habitat. However, human practices are also responsible for the expansion of pathogens' geographic area. In particular, multiple crop epidemics were caused by multiple introductions pathogen populations from restricted geographic regions, as reported for the worldwide spread of kiwifruit bacterial canker caused by the bacterium *Pseudomonas syringae* pv. *actinidiae* originating from China (Kim et al., 2016) and for *P. infestans* (Kamoun et al., 2015).

The challenge of understanding and predicting ED occurrences is even more relevant in the climate change context which is likely to favor conditions for pathogens' development and dispersal (Bergot et al., 2004; Garrett et al., 2006; Tylianakis et al., 2008). Consequently, climate change scenarios predict an increase in the number of epidemics in the next coming decades (Bergot et al., 2004; Evans et al., 2008). However, the relationships between climate change and ED occurrences is also related to the life histories and the infection processes of the pathogens. For example, pathogens living in highly fluctuating environments (such as epiphytes) should be more favored under a climatic change scenario than pathogens strictly adapted to more constant habitats (such as obligate endophytes). Taking into account the environmental drivers of the pathogen/host variability that is the central theme for ED occurrence, there is an increasing need to better identify and understand the genetic and molecular mechanisms underlying pathogen virulence and plant resistance in the ecological conditions where both pathogens and hosts evolve. To our opinion, this ecological genomics approach can favor the identification of novel, durable, and sustainable means to prevent crop diseases.

To date, traditional linkage mapping based on genetic map, has been achieved in the identification of the genetic basis underlying phenotypic variation in both plants and pathogenic microbes. Traditional linkage mapping refers to a diversity of experimental populations ranging from F2 populations to the more recently developed multiparent advanced generation intercross (MAGIC) lines (Kover et al., 2009). Because Recombinant Inbred Lines (RILs) are almost completely homozygous, they can be replicated within an experiment and/or among several environmental conditions, thereby making RIL families the most popular experiment populations for traditional linkage mapping, at least in plants (Bergelson and Roux, 2010). While few genetic markers are required for a complete genome scan, traditional linkage mapping presents several drawbacks including (i) coarse mapping, (ii) genetic diversity that is limited to the parental lines of the segregating populations, and (iii) the impossibility to distinguish between pleiotropic and physically close genes (Bergelson and Roux, 2010). To address those problems, the method of genome-wide association (GWA) mapping emerged through the recent development of next-generation sequencing (NGS) technologies. Taking advantage of recombination events that have accumulated over thousands of generations (Mitchell-Olds and Schmitt, 2006; Nordborg and Weigel, 2010), GWA mapping uses natural linkage disequilibrium (LD) to identify polymorphisms that are associated with phenotypic variation. Although its power is reduced to detect rare alleles or weak-effect alleles in GWA mapping populations, this issue is counterbalanced by the greater advantage of fine mapping (down to the gene level) and common alleles associated with phenotypic variation at the species level (Bergelson and Roux, 2010).

The aim of the present review is to summarize recent progresses on the identification of QTLs underlying plant-pathogen interactions, through the use of GWA mapping. We discussed the main experimental and methodological difficulties that need to be addressed in the future to obtain a thorough overview of the genetic architecture underlying plant-pathogen interactions. We also introduced the emerging joint GWA mapping approach that allows the simultaneous identification of intergenomic epistatic QTLs underlying the molecular landscape of plant-pathogen interactions. In this context, we highlighted the benefits of the joint GWA mapping approach, in particular when performed in absence of phenotyping.

## GWA studies of pathogen resistance in plants

In this review, we only considered GWAS based on a substantial number of genetic markers covering the whole plant species genome, thereby allowing to obtain an unbiased picture of the genetic architecture driving disease resistance. Also, we focused on studies where plants were directly challenged with a pathogen species. We therefore not considered GWAS of autoactive hypersensitive response (Olukolu et al., 2014). Based on these criteria, we identified a total of 35 studies reporting the identification of genomic regions associated with natural variation of plant response to pathogen infection in 34 pathosystems (Table 1). Despite the limited number of GWAS of disease resistance, important observations can be still drawn. Firstly, although the first GWAS of disease resistance—on *Arabidopsis thaliana* interacting with the bacterial pathogen *P. syringae*—was published in 2005 (Aranzana et al., 2005), almost half of the GWAS (~48.6%) have been published in the last 2 years. The recent burst of GWAS is directly linked to the development of the NGS technologies that occurred in the last decade. NGS technologies provide sufficient numbers of genetic markers to fine-map genes underlying natural variation of complex traits (Bergelson and Roux, 2010). The 35 GWAS identified here used Single Nucleotide Polymorphisms (SNPs) as genetic markers for mapping, because of their high frequency across the genomes and the development of SNP-tilling arrays containing probe sets for tens (even hundreds) of thousands of SNPs. While SNP markers remain highly popular, they represent only a fraction of the available genetic polymorphisms. The access to structural variants such as copy number variation (CNV) and insertions-deletions (indels) is already facilitated by Single Molecule Real-Time (SMRT) sequencing technologies such as the Pacific Biosciences (PacBio) and Oxford Nanopore systems (Goodwin et al., 2016; Lee et al., 2016). In addition, because epigenetic variation can account for a non-negligible fraction (up to 30%) of the variation in complex traits (Roux et al., 2011), epigenome characterization at a single-base-pair resolution for hundreds of plant lines has already started for a limited number of plant species. For example, a high-quality single-base resolution genome-wide methylome was recently reported for 1,107 natural *A. thaliana* accessions (Kawakatsu et al., 2016). While combining different types of genomic markers with epigenomic diversity should help the access to causal variations and to tease apart the relative effect of genetic variants from epigenetic variants, the inclusion of additional information ever lead to an increase of false-positive associations between phenotype and polymorphic markers due to the problem of large dimensionality. The reduction of false-positive rate requests the development of statistical methods taking into account the diversity of polymorphic markers and the inherent kinships among plant lines, potentially reflecting different aspects of the demographic history of the plant species considered.

**Table 1:** **Genome-wide association studies in plant pathosystems**.

	**Plant species name**	**Number of lines^a^**	**Polymrophisms^b^**	**Pathogen species name**	**Phenotypic trait^c^**		**Growth conditions^d^**	**Gen. Arch.^e^**	**Functional validation^f^**	**References**
Bacteria	*Arabidopsis thaliana*	84 NA	>17 k SNPs	*Pseudomonas syringae—*Pst DC3000::avrRpm1	B	Hypersensitive response	G	M	*RPM1*	Aranzana et al., 2005
		87 NA	>17 k SNPs	*Pseudomonas syringae—*Pst DC3000::avrB	B	Hypersensitive response	G	M	*RPM1*	Aranzana et al., 2005
		90 NA	>17 k SNPs	*Pseudomonas syringae—*Pst DC3000::avrPphB	B	Hypersensitive response	G	M	*RPS5*	Aranzana et al., 2005
		89 NA	>17 k SNPs	*Pseudomonas syringae—*Pst DC3000::avrRpt2	B	Hypersensitive response	G	M	*RPS2*	Aranzana et al., 2005
		84 NA	>216 k SNPs	*Pseudomonas syringae—*Pst DC3000::avrRpm1	B	Hypersensitive response	G	M	*RPM1*	Atwell et al., 2010
		87 NA	>216 k SNPs	*Pseudomonas syringae—*Pst DC3000::avrB	B	Hypersensitive response	G	M	*RPM1*	Atwell et al., 2010
		90 NA	>216 k SNPs	*Pseudomonas syringae—*Pst DC3000::avrPphB	B	Hypersensitive response	G	M	*RPS5*	Atwell et al., 2010
		89 NA	>216 k SNPs	*Pseudomonas syringae—*Pst DC3000::avrRpt2	B	Hypersensitive response	G	M	*RPS2*	Atwell et al., 2010
		95 NA	>216 k SNPs	*Pseudomonas syringae—*Pst DC3000	Q	Bacterial growth	G	P	No	Atwell et al., 2010
		96 NA	>205 k SNPs	*Pseudomonas syringae—*Pst DC3000	Q	Bacterial growth	G	P	*AtABCG36*	Ji et al., 2014
		64 NA	4,004,754 SNPs	*Pseudomonas syringae—*Pst DC3000D28E (virulence factor HopAM1)	Q	DI (time point that cell death was first observed)	G	P	No	Iakovidis et al., 2016
					B	Chlorotic rosette phenotype	G	P	No	Iakovidis et al., 2016
		75 NA	>216 k SNPs	*Pseudomonas syringae—*natural strain PNA29.1a	B	Hypersensitive response	G	M	*RPS5*	Karasov et al., 2014
		175 NA	>216 k SNPs	*Pseudomonas viridiflava—*natural strain LP23.1a	Q	DI (chlorosis) and bacterial growth	G	P	No	Atwell et al., 2010
		175 NA	>216 k SNPs	*Pseudomonas viridiflava—*natural strain RMX23.1a	Q	DI (chlorosis) and bacterial growth	G	P	No	Atwell et al., 2010
		175 NA	>216 k SNPs	*Pseudomonas viridiflava—*natural strain RMX3.1b	Q	DI (chlorosis)and bacterial growth	G	P	No	Atwell et al., 2010
		175 NA	>216 k SNPs	*Pseudomonas viridiflava—*natural strain PNA3.3a	Q	DI (chlorosis) and bacterial growth	G	P	No	Atwell et al., 2010
		175 NA	>216 k SNPs	*Pseudomonas viridiflava—*natural strain ME3.1b	Q	DI (chlorosis)and bacterial growth	G	P	No	Atwell et al., 2010
		163 NA	>214 k SNPs	*Ralstonia solanacearum—*crop strain GMI1000	Q	DI (wilting)	G	P	*RRS1*/*RPS4 & SSL4*	Aoun et al., in review
		384 NA	>214 k SNPs	*Xanthmonas campestris pv*. *campestris—*crop strain *Xcc568*	Q	DI (chlorosis)and bacterial growth	G	P	*RKS1*	Huard-chauveau et al., 2013
	*Arabidopsis thaliana*	384 NA	>214 k SNPs	*Xanthmonas campestris pv*. *campestris—*crop strain *Xcc12824*	Q	DI (chlorosis)	G	P	*AT5G22540*	Debieu et al., 2016
		176 NA	>214 k SNPs	*Xanthmonas campestris pv*. *campestris—*crop strain *XccCFBP6943*	Q	DI (chlorosis)	G	P	*RRS1*/*RPS4*	Debieu et al., 2016
	*Glycine max*	3,173 Accessions	37,659 SNPs	*Xanthomonas axonopodis* pv. *glycines*	Q	Di (percentage of lesions)	F (NE)	P	No	Chang et al., 2016
Fungi	*Arabidopsis thaliana*	96 NA	115,301 SNPs	*Botrytis cinerea—*strain Apple 517	Q	Lesion area + camalexin production	G	P	Yes	Corwin et al., 2016
		96 NA	115,301 SNPs	*Botrytis cinerea—*strain B05.10	Q	Lesion area + camalexin production	G	P	Yes	Corwin et al., 2016
		96 NA	115,301 SNPs	*Botrytis cinerea—*strain Supersteak	Q	Lesion area + camalexin production	G	P	Yes	Corwin et al., 2016
		96 NA	115,301 SNPs	*Botrytis cinerea—*strain UKRazz	Q	Lesion area + camalexin production	G	P	Yes	Corwin et al., 2016
		350 NA	>214 k SNPs	*Botrytis cinerea*	Q	Percentage of leaves with spreading lesions	G	P	No	Thoen et al., 2016
		350 NA	>214 k SNPs	*Botrytis cinerea—*strain B05.10	Q	*rosette fresh weight*	G	P	No	Davila Olivas et al., 2016
	*Brassica napus*	179 Diverse accessions	18,804 SNPs	*Leptosphaeria maculans—*12 single spore isolates (04MGPS021, 06MGPP041, D8, D9, IBCN13, IBCN15, IBCN16, IBCN17, IBCN18, IBCN75, IBCN76 and PHW1223)	Q	DI (lesion size)	G	P	No	Raman et al., 2016
		179 Diverse accessions	18,804 SNPs	*Leptosphaeria maculans—*mix of pathotypes from four canola stubble sources	Q	Percentage of internal canker infection	G	P	No	Raman et al., 2016
		116 Varieties	4,239 SNS	*Leptosphaeria maculans*	Q	DI (area of necrosis)	F (NE)	P	No	Fopa Fomeju et al., 2014
	*Glycine max*	392 Diverse cultivars + 300 advanced breeding lines	52,041 SNPs + 5,361 SNPs	*Fusarium virguliforme*	Q	DI (disease incidence ^*^ disease severity/9)	F (NE)	P	No	Wen et al., 2014
		214 Germplasm accessions	31,914 SNPs	*Fusarium virguliforme—*isolate Mont-1	Q	DI (percentage of foliage affected) + disease progressive curve	G	P	No	Zhang et al., 2015
		4,771 Accessions	37,991 SNPs	*Fusarium virguliforme—*isolate Mont-1	Q	DI (percentage of foliage affected)	G	P	No	Chang et al., 2016
		130 Lines	7,864 SNPs	*Sclerotina sclerotorium—*strain NB5	Q	Lesion length	G	P	No	Bastien et al., 2014
Fungi	*Glycine max*	2773 Accessions	33,240 SNPs	*Cadophora gregata*	B	Combination of foliar and stem observations	F (NE)	P	No	Rincker et al., 2016
		540 Accessions	33,486 SNPs	*Cadophora gregata*	Q	Percentage of plants exhibiting symptoms + proportion of nodes exhibiting brown pith	F (NE)	NA	No	Rincker et al., 2016
		825 Accessions	32,150 SNPs	*Cadophora gregata—*strain Oh2	Q	Proportion of plants exhibiting foliar symptoms	G	P	No	Rincker et al., 2016
		606 Accessions	29,815 SNPs	*Cadophora gregata—*strain Oh2	Q	Proportion of nodes exhibiting brown pith	G	P	No	Rincker et al., 2016
		608 Accessions	34,424 SNPs	*Cadophora gregata*	Q	Four classes of phenotypes based on foliar and stem symptoms	G + F (NE)	P	No	Chang et al., 2016
		1,426 Accessions	33,549 SNPs	*Diaporthe phaseolorum* var. *caulivora*	Q	DI (length of the visible lesions + proportion of dead plants)	G	P	No	Chang et al., 2016
		112 accessions	34,210 SNPs	*Diaporthe phaseolorum* var. *meridionalis*	Q	DI (percentage of infected/dead plants)	G	P	No	Chang et al., 2016
		2,385 Accessions	38,608 SNPs	*Phakopsora pachyrhizi—*mixture of four isolates	Q	DI (symptom and lesion development) + type of lesion	G	P	No	Chang et al., 2016
	*Hordeum vulgare*	92 Commercial cultivars	6,970 SNPs	*Puccina graminis—*mixture of isolates representative of the most prevailing races of the pathogen in Central Asia	Q	DI (uredinia size, chlorosis and necrosis)	F (AI)	P	No	Turuspekov et al., 2016
	*Medicago sativa*	179 Elite breeding lines	19,801 SNPs	*Verticillium alfalfae—*mix of six strains (Berg, Sevcik, Freitag, Kunderts, VP6 and VP11)	Q	DI (severity of disease symptoms)	G	P	No	Yu et al., 2016
	*Oryza sativa*	413 Cultivars	44,100 SNPs	*Pyricularia oryzae—*mix of US races IB-49, IC-17 and IE-1K	Q	DI (lesions)	F (AI)	P	No	Zhao et al., 2011
		362 cultivars	700 k SNPs	*Magnaporthe oryzae—*isolate R01-1	Q	DI (lesion area)	G	P	*Pi5 locus*	Kang et al., 2016
		331 Cultivars	700 k SNPs	*Magnaporthe oryzae—*isolate RB22	Q	DI (lesion area)	G	P	*Pi5 locus*	Kang et al., 2016
		312 Cultivars	700 k SNPs	*Magnaporthe oryzae—*isolate 75-1-127	Q	DI (lesion area)	G	P	*Pi5 locus*	Kang et al., 2016
		335 Cultivars	700 k SNPs	*Magnaporthe oryzae—*isolate 0-249	Q	DI (lesion area)	G	P	*Pi5 locus*	Kang et al., 2016
		333 Cultivars	700 k SNPs	*Magnaporthe oryzae—*isolate P06-6	Q	DI (lesion area)	G	P	*Pi5 locus*	Kang et al., 2016
	*Setaria italica*	916 Varieties (traditional landraces + modern cultivars)	845,787 SNPs	*Magnaporthe grisea*	Q	not available	F (NE)	P	No	Jia et al., 2013
				*Rhizoctonia solani*	Q	not available	F (NE)	P	No	Jia et al., 2013
				*Uromyces setariae-italicae*	Q	not available	F (NE)	P	No	Jia et al., 2013
	*Sorghum bicolor*	300 Genotypes (251 tropical sorghums + 49 breeding lines)	79,132 SNPs	*Macrophomina phaesolina*	Q	Length of the visible lesions	F (AI)	P	No	Adeyanju et al., 2015
				*Fusarium thapsinum*	Q	Length of the visible lesions	F (AI)	P	No	Adeyanju et al., 2015
	*Zea mays*	4,699 RILs (NAM population)	1.6 M SNPs	*Cochliobus heterostrophus*	Q	DI (number and area of lesions)	F (NE + AI)	P	No	Kump et al., 2011
		4,413 RILs (NAM population)	28.5 M genomic variants (SNPs + read-depth variants)	*Cochliobus heterostrophus*	Q	DI (number and area of lesions)	F (NE + AI)	P	No	Bian et al., 2014
		4,630 RILs (NAM population)	1.6 M SNPs	*Setosphaeria turcica—*race 1	Q	Percentage of diseased leaf area	F (AI)	P	No	Poland et al., 2011
		3,678 RILs (NAM population)	1.6 M SNPs	*Cercospora zeae-maydis* + *Cercospora zeina*	Q	DI (dimension of each lesion + number of lesions)	F (NE)	P	No	Benson et al., 2015
		1487 European inbred lines	8,244 SNPs	*Setosphaeria turcica*	Q	DI (Percentage of diseased leaf area)	F (NE + AI)	P	No	Van Inghelandt et al., 2012
		144 Inbred lines	45,868 SNPs	*Sphacelotheca reiliana—*spores collected from the field in the previous growing season	Q	Percentage of infected plants	F (AI)	P	No	Wang et al., 2012
		267 Inbred lines	47,445 SNPs	*Fusarium verticilliodes—*local isolates	Q	Percentage of the ear presenting disease symptoms + DI (percentage of kernels exhibiting visual symptoms of infection)	F (AI)	P	No	Zila et al., 2013
		274 Inbred lines	246,497 SNPs	*Puccina sorghi—*mix of races of spores collected during the previous season	Q	DI (scale of rust infection on the plant)	F (AI)	P	No	Olukolu et al., 2016
Oomycete	*Arabidopsis thaliana*	86 NA	>216 k SNPs	*Hyaloperonospora arabidopsidis—*natural strain Emco5	B	Presence/absence of sporangiophores	G	P	*RPP13,RPP5*	Atwell et al., 2010; Nemri et al., 2010
		85 NA	>216 k SNPs	*Hyaloperonospora arabidopsidis—*natural strain Emwa1	B	Presence/absence of sporangiophores	G	P	No	Atwell et al., 2010; Nemri et al., 2010
		76 NA	> 216k SNPs	*Hyaloperonospora arabidopsidis—*natural strain Emoy2	B	Presence/absence of sporangiophores	G	P	*RPP13*	Atwell et al., 2010; Nemri et al., 2010
	*Arabidopsis thaliana*	84 NA	>216 k SNPs	*Hyaloperonospora arabidopsidis—*natural strain Hiks1	B	Presence/absence of sporangiophores	G	P	*RPP7*	Atwell et al., 2010; Nemri et al., 2010
		87 NA	>216 k SNPs	*Hyaloperonospora arabidopsidis—*natural strain Noco2	B	Presence/absence of sporangiophores	G	P	*RPP5, RPP7*	Atwell et al., 2010; Nemri et al., 2010
	*Glycine max*	~879 Accessions (range from 44 to 7,431)	~33,709 SNPs (range from 24,490 to 41,911)	*Phytophthora sojae—*19 individual races	Q	DI (proportion of alive plants or plants with non-killing lesions; three classes)	G	P	No	Chang et al., 2016
	*Medicago truncatula*	179 NA	~5.1 M SNPs	*Aphanomyces euteiches—*strain ATCC 201684	Q	Porportion of brown symptomatic tissues on roots and stem + amounts of cotyledon yellowing + proportion of dead plants + DI (discoloration of the roots)	G	P	No	Bonhomme et al., 2014
	*Pisum sativum*	175 Lines (cultivars + breeding lines + germplasm lines)	13,204 SNPs	*Aphanomyces euteiches—*French strain RB84	Q	DI (Percentage of browning symptoms on roots and epicotyls)	G	P	No	Desgroux et al., 2016
			13,204 SNPs	*Aphanomyces euteiches—*American strain Ae109	Q	DI (Percentage of browning symptoms on roots and epicotyls)	G	P	No	Desgroux et al., 2016
			13,204 SNPs	*Aphanomyces euteiches*	Q	DI (Percentage of browning symptoms on roots and epicotyls + yellowing symptoms on a plot)	F (NE)	P	No	Desgroux et al., 2016
	*Setaria italica*	916 Varieties (traditional landraces + modern cultivars)	845,787 SNPs	*Sclerospora graminicola*	Q	Not described	F (NE)	P	No	Jia et al., 2013

a *Number of genetic lines that have been phenotyped for pathogen response. NA: natural accessions*.

b *SNPs: Single Nucleotide Polymorphisms*.

c *“B” and “Q” stand for binary and quantitative traits, respectively. DI: disease index (generally based on a scale from 0 to 9)*.

d *G, greenhouse conditions; F, field conditions; NE, natural exposition to the pathogen; AI, artificial inoculation*.

e *Genetic architecture. M, monogenic; P, polygenic; NA, not available*.

f *Name of the genes underlying QTLs that have been functionally validated. Genes in red indicate genes that have been functionally validated after QTL detection by GWA mapping*.

Secondly, the 35 plant GWAS are not evenly distributed across the three classes of pathogenic organisms considered in this review (Table 1). Two thirds of the GWAS reported the mapping of resistance QTLs to fungal pathogens, whereas the remaining GWAS were split between bacterial pathogens (*n* = 9) and oomycete pathogens (*n* = 6). The greater number of GWAS designed to identify genes decreasing the detrimental effect of fungal infection on plants is in line with fungal pathogens being the most widespread and rapidly spreading crop pathogens, despite their restricted host range in comparison with bacterial and oomycete pathogens (Bebber et al., 2014). Although plants are simultaneously and/or sequentially attacked by a range of pathogens, whether in natural environments or in crop fields (Kniskern et al., 2007; Davila Olivas et al., 2016; Roux and Bergelson, 2016), GWAS reporting genomic regions associated with plant responses to multiple pathogen attacks remain however scarce. In a first attempt, by measuring resistance to three fungal leaf diseases (i.e., southern leaf blight caused by *Cochliobolus heterostrophus*, gray leaf spot caused by *Cercospora zeae-maydis* (Czm) and northern leaf blight caused by *Cercospora zeina*) on 253 maize inbred lines genotyped for only 858 SNPs (i.e., 0.0015% SNP sites in maize), Wisser et al. (2011) performed a multivariate analysis and identified a glutathione *S*-transferase (*GST*) associated with modest levels of resistance to all three diseases. The recent development of statistical tools to perform multi-trait GWA mapping would undoubtedly facilitate the identification of the pleiotropic genetic determinants underlying multi-pathogen response (Korte et al., 2012; Thoen et al., 2016). In the era of metagenomics, describing multiple pathogen infections can be facilitated by the description of the pathobiota (defined as the complex of microorganisms with the potential to cause disease on the plant host; Kamada et al., 2013) based on high throughput sequencing of amplicons of housekeeping genes with deep taxonomic resolution (such as *gyrB* gene marker for bacteria), allowing to distinguish pathogenic from commensal Operational Taxonomic Units (OTUs) within a given microbe genus (Barret et al., 2015). A metagenomic approach can help to estimate the relative fraction of microbial pathogens and can also have the advantage of describing multiple pathogen infections at very early disease stages. Under these considerations, the next challenge in tracing the plant genomic regions associated with disease occurrence concerns the developed of GWAS on the whole pathobiota. While a recent GWA study reported the identification of genomic regions associated with descriptors (i.e., species richness, α-diversity, β-diversity, presence/absence of OTUs) of leaf microbial community in *A. thaliana* (Horton et al., 2014), to our knowledge, no GWAS on descriptors of pathobiota communities have been published so far. Altogether, mapping genomic regions associated with plant response to multiple pathogen infections can help to better elucidate the pathways that ultimately enable a plant to fine-tune its defense against different aggressors, thereby shedding some light on downstream components of the complex signaling network leading to resistance that cannot be revealed by classical mono-pathogen infection approaches.

Thirdly, the 35 GWAS involved only 11 plant species distributed across three botanical families, i.e., Brassicaceae (*A. thaliana* and *Brassica napus*), Fabaceae (*Glycine max, Medicago sativa, Medicago truncatula, Pisum sativum*) and Poaceae (*Hordeum vulgare, Oryza sativa, Setaria italica, Sorghum bicolor*, and *Zea mays*; Table 1). Because of the economic and environmental cost of crop pathogens, the majority of GWAS (~62.8%) was conducted on crop species to speed up the identification of new sources of disease resistance. The remaining GWAS were conducted on the two model plant species *A. thaliana* (~33%) and *M. truncatula* (~3%). Conducting GWAS on wild plant species can be a successful starting point to identify homologous genes in other species belonging or not to the same botanical family (Huard-chauveau et al., 2013). In addition, studying the spatial-temporal evolutionary dynamics of an adaptive resistance gene in an ecologically realistic context can help drawing new strategies for disease management in crops in an agro-ecological context (Bergelson et al., 2001; Barrett et al., 2009; Burdon et al., 2014; Karasov et al., 2014; Roux et al., 2014a).

Fourthly, a large variety of genetic lines, from natural accessions to elite breeding lines, were used for scoring disease resistance (Table 1). Noteworthy is the use of the combined advantages of both traditional QTL mapping and GWA mapping in a Nested Association Mapping (NAM) population in maize (for a total of ~4,000 RILs resulting from 25 crosses between diverse inbred lines and the reference line B73; Buckler et al., 2009) in four GWAS (Kump et al., 2011; Poland et al., 2011; Bian et al., 2014; Benson et al., 2015). Across the 35 GWAS, the number of genetic lines scored for disease resistance follows an L-shaped distribution with a median of ~196 lines (mean number ~817 lines, minimum number ~44 lines, maximum number ~7,431 lines; Table 1). Increasing genetic diversity remains the major goal in the design of a GWA mapping population and is mainly achieved by assembling lines collected over the entire geographic range of a plant species. Such panels may however increase the effect of population demographic history on the rate of false-positive phenotype-genotype associations (Zhao et al., 2007). Statistical methods controlling for population structure can drastically reduce the inflation of false-positive associations (Price et al., 2006, 2010; Kang et al., 2010; Zhang et al., 2010), but with the cost of increasing the rate of false-negative associations (i.e., when causative variants are lost after applying a correction for the effects of population structure; Brachi et al., 2010). In addition, because the same phenotype can be achieved by different combinations of genes, a higher genetic diversity increases the probability of genetic and/or allelic heterogeneity which may in turn limit the detection of polymorphic markers linked to phenotypic variation (Bergelson and Roux, 2010). Controlling the effects of genetic and allelic heterogeneity may be achieved by building panels of lines belonging to the same genetic cluster that is often geographically restricted. While of smaller size, those panels can lead to a higher power and resolution to fine-map loci associated with phenotypic variation. For example, more significant and neat association peaks for phenological variation in *A. thaliana* were found in set of 121 natural accessions collected in the region of Burgundy (France) than in a set a 167 worldwide natural accessions (Brachi et al., 2013). It remains to be tested whether a similar pattern is also observed for disease resistance.

Fifthly, the environmental conditions in which phenotyping of disease resistance was performed are well balanced between laboratory (greenhouse/growth chamber) controlled conditions (60%) and field conditions (40%) (Table 1), with only three GWAS performed both in controlled and/or field conditions (Chang et al., 2016; Desgroux et al., 2016; Rincker et al., 2016). Controlled and field conditions are complementary. In the field, plants are exposed to greater temporal abiotic fluctuations that may affect plant responses to pathogen invasions than are typically encountered in laboratory conditions. To limit the effects of those fluctuations, field experiments are repeated over several years. Despite recurrent observations of pathogen infections in natural populations, it is interesting to note that no GWAS was performed in the model plant species *A. thaliana* and *M. truncatula* in their local habitats. Adding ecology to the studies of “disease resistance—genotype” association can however help to better understand the evolutionary trajectories of a given adaptive resistance gene (Karasov et al., 2014; Roux and Bergelson, 2016). On the other hand, performing experiments in controlled conditions can help to test the effect of a specific abiotic stress on plant resistance to pathogen infection. This is especially relevant in the context of climate change where (i) the severity of epidemics is predicted to increase due to the shift and broadening of geographic distributions of pathogen species (Evans et al., 2008; Bebber et al., 2014), and (ii) a permanent increase in temperature was demonstrated to alleviate major known defense mechanisms in plants (Suzuki et al., 2014). In addressing this issue, Aoun and colleagues reported a GWAS performed on *A. thaliana* challenged with the bacterial pathogen *Ralstonia solanacearum* at 27 and 30°C (Aoun et al., in review). Based on traditional QTL mapping performed on one RIL family, the immune receptor pair of TIR-NBS-LRR proteins RESISTANT TO *P. SYRINGAE* 4 (RPS4)/RESISTANT TO *R. SOLANACEARUM* 1 (RRS1) has been map-based cloned and identified as the major genetic determinant conferring resistance to the *R. solanacearum* GMI1000 strain at 27°C (Deslandes et al., 2002). GWA mapping performed on 176 worldwide accessions of *A. thaliana* phenotyped at 27°C revealed a strong and unique association peak with the most associated SNP located within *RPS4*, suggesting that natural variation for resistance to the strain GMI1000 is also caused by *RPS4*/*RRS1* at the species level (Aoun et al., in review). At 30°C, GWA mapping performed on the same set of accessions revealed multiple and smaller association peaks not located in the vicinity of *RPS4*/*RRS1*. Based on the phenotyping of T-DNA knockout mutants, the authors identified an enzyme encoding a strictosidine synthase (*STRICTOSIDINE SYNTHASE-LIKE 4, SLL4*) as underlying one of these small QTLs (Aoun et al., in review).

Sixthly, the first GWAS of disease resistance were performed in *A. thaliana* on natural variation of the hypersensitive response (HR), i.e., a highly effective local resistance response that is often associated with hypersensitive cell death (Table 1). This binary trait was useful to demonstrate the power of GWA mapping in plants because of previously known resistance genes underlying HR (Aranzana et al., 2005; Atwell et al., 2010). However, quantitative resistance (continuum of symptoms) is much more prevalent than qualitative resistance (presence/absence of symptoms) in crops and natural plant populations (Young, 1996; Poland et al., 2008; Roux et al., 2014a). Accordingly, recent GWAS performed in *A. thaliana* and *M. truncatula* and all GWAS performed in crops were based on a quantitative scoring of plant response to pathogen infections (but see Rincker et al., 2016; Table 1). The quantitative genetic architecture was highly diverse among pathosystems, ranging from the identification of few medium-effect QTLs to the identification of up to hundreds (and even thousands) of small-effect QTLs (Corwin et al., 2016). We should however be cautious on the complexity of the quantitative genetic architecture described in most GWAS because the number of QTLs identified might be highly dependent on the number of genetic lines used, the number of polymorphic markers genotyped and the accuracy in scoring the disease symptoms (Table 1). Because the development of NGS technologies will speed up the accumulation of genomic resources, the next frontier is high-throughput phenotyping of precise quantitative disease symptoms. This challenge can be achieved by the combination of the development of automated platforms (such as the Toulouse Plant Microbe Phenotyping Platform) updated by the International Plant Phenomics Network (IPPN) with the development of image-based quantification of disease symptoms (Laflamme et al., 2016).

Finally, the major goal of performing GWA mapping in crops is to accelerate the identification of genetic markers that can be subsequently used for Marker-Assisted Selection (MAS) in breeding programs. In this context, GWAS are rarely followed up by studies aiming at functionally validating the causative genes. To date, after identification of genomic regions linked to disease resistance by GWA mapping, genes responsible for the QTL of interest were functionally validated in only six GWAS (Table 1). Unsurprisingly, most functional validations were performed in *A. thaliana* due to the impressive genetic tools available in this species, such as the availability of several public collections of T-DNA mutants, quantitative complementation (i.e., introducing alternative alleles in genetic lines lacking the candidate gene) or quantitative knockdown (i.e., gene silencing by amiRNA; Bergelson and Roux, 2010). Functional validation helped (i) to identify gene functions that have never been related to defense against aggressors, as illustrated by the atypical kinase *RKS1* gene and a gene of unknown function, both conferring quantitative resistance to the bacterial pathogen *Xanthomonas campestris* (Huard-chauveau et al., 2013; Roux et al., 2014b; Debieu et al., 2016), and (ii) to establish the selective forces acting on the causative gene (Huard-chauveau et al., 2013; Karasov et al., 2014). In a further step, both analyzing the transcriptional and/or post-transcriptional regulation of the causative gene and searching for proteins directly interacting with the causative gene can lead to the identification of the downstream signaling pathways, thereby providing an additional list of candidate genes for breeding programs.

## Why so few GWAS of pathogenicity in bacteria, fungi and oomycetes?

Similarly to plants, we only considered in this study GWAS based on a substantial number of genetic markers covering the whole genome of the pathogen species. As described above, GWA mapping started to be extensively used to fine map resistance genes in plants. By contrast, since the publication of the first plant pathogenic bacterium genome (*Xylella fastidiosa*) in 2000 (Simpson et al., 2000), the first plant pathogenic fungal species *Magnaporthe grisea* in 2005 (Dean et al., 2005) and the first oomycete species *Phytophthora ramorum* in 2006 (Tyler et al., 2006), as well the establishment of the Fungal Genome Initiative (FGI) (Galagan et al., 2005), only five studies employed GWA mapping to identify candidate pathogenic genetic determinants in bacterial (Monteil et al., 2017) and fungal pathogens (Dalman et al., 2013; Gao et al., 2016; Talas et al., 2016, Wu et al., 2017) and to our knowledge, no GWAS was reported yet on a phytopathogenic oomycete. Several explanations can be advanced for this paucity of studies. Firstly, comparative genomics has been proved as a very efficient tool to identify important pathogenic genetic determinants and to elucidate the mechanisms that microbial phytopathogens employ during pathogenesis (Klosterman et al., 2016). As recently summarized by Sundin et al. (2016), comparative genomics performed on an ever-growing number of phytopathogenic sequenced genomes was extremely useful for the discovery of type III secretion system (T3SS) effectors and transcription activator-like effector nuclease (TAL-effector) of the three most important studied plant pathogenic bacteria *P. syringae, X. campestris* and *R. solanacearum* (Mansfield et al., 2012). Though powerful, comparative genomics has also several limitations. Comparative genomics is a powerful tool to compare closely related epidemic strains showing few genomic differences but it can be hardly applied to “reservoir populations” carrying a high degree of diversity among them. In addition, comparative genomics is mainly employed to detect phenotypic differences that are based on the presence/absence of genes, thereby strongly limiting the potential for identifying SNPs associated with natural variation of virulence/aggressiveness among microbial pathogenic populations.

Secondly, despite the presence of several molecular mechanisms that can generate genetic variation in microbes (i.e., frequent mutation events, homologous recombination, and horizontal gene transfer; Bartoli et al., 2016), fine mapping in GWAS can be limited by the long LD observed in many pathogen species (Read and Massey, 2014). In human pathogenic microbes, long LD is mainly observed in pathogen species with a clonal reproduction system and/or in sets of epidemics strains with limited genetic diversity (Chen and Shapiro, 2015; Read and Massey, 2014). While to our knowledge no LD extent has been estimated in phytopathogenic bacteria and oomycetes, a rapid LD decay was observed in two phytopathogenic fungi, i.e., *Fusarium graminearum* (mean LD ~1 kb) and *Parastagonospora nodorum* (mean LD ~5–10 kb; Gao et al., 2016; Talas et al., 2016), thereby allowing fine mapping of pathogenic determinants (see below).

Thirdly, as previously observed in plants, genomic diversity of pathogenic microbes can be strongly shaped by population stratification (Power et al., 2017). Such population stratification is particularly encountered in haploid and asexual bacteria and/or pathogenic microbes with limited dispersal (Chen and Shapiro, 2015). Because population structure can impede the identification of genomic regions associated with virulence/aggressiveness in pathogenic microbes, various methods have been recently developed to limit the rate of false positives (Sheppard et al., 2013; Earle et al., 2016). When applied in the field of human pathology, these methods allowed the identification of genes related to host specificity in the *Campylobacter* human pathogen (Sheppard et al., 2013) and genes associated with antibiotic resistance in both *Mycobacterium tubercolosis* and *Streptococcus* bacterial species (Read and Massey, 2014).

To limit the negative effects of long LD and population structure on the identification of pathogenic genetic determinants, we advise the use of a local/ regional set of microbial strains collected on wild plant species. Such a strategy should increase the level of genetic diversity that is available in the natural plant reservoirs, while limiting the problem of population stratification.

If the last 4 years are documented by a speed in the increasing of studies employing GWAS to detect genes important for pathogenicity in human pathogens (Power et al., 2017), GWA mapping is still poorly used in plant pathology to identify genes related to microbial pathogenicity phenotypes. To our knowledge, the study from Monteil et al. (2017) is the only one that attempted to apply GWA mapping to a phytopathogenic bacterium. By using a GWA mapping method previously developed for human bacterial pathogens and that takes into account both core and pan-genome while controlling for population structure (Sheppard et al., 2013; Pascoe et al., 2015; Yahara et al., 2017), the authors found that the T3SS effectors *hopQ1* and *hopD1* have probably shaped the adaptation of the ubiquitous plant pathogenic bacterium *P. syringae* to crops. In plant-fungus pathosystems, we identified four studies reporting the identification of genetic determinants associated with virulence/aggressiveness. Dalman et al. (2013) adopted a GWA mapping approach to identify the genetic components underlying virulence in the fungal necrotrophic pathogen *Heterobasidion annosum sensu stricto* that is responsible of severe damages in forest conifers. Based on 23 haploid whole-genome sequenced *H. annosum* isolates collected in different geographic European countries, the authors used 33,018 non-singleton SNPs to run GWA mapping on virulence scored on both Scots pine and Norway spruce in controlled conditions. Although the size of the mapping population was limited, 12 SNPs were found to be significantly associated with virulence on both host plants. In the study of Talas et al. (2016), 119 isolates of the fungal pathogen *F. graminearum* collected in Germany were phenotyped for aggressiveness on wheat under field conditions in two locations over 2 years (Talas et al., 2016). Based on ~29,000 SNPs and a short LD of <1 kb, the authors finely mapped 50 SNPs significantly associated with aggressiveness. Interestingly, highly significant interactions between the isolates and the field phenotyping conditions suggested an environment-dependent genetic architecture of *F. graminearum*. In the study of Gao et al. (2016), 191 isolates of the fungal necrotrophic wheat pathogen *P. nodorum* were phenotyped for virulence on two wheat lines and genotyped for ~3,000 SNPs distributed across the genome as well as genetic markers in candidate genes. The identification of the two previous cloned effector genes *SnToxA* and *SnTox3* confirmed the power of GWA mapping to fine map virulence factors in *P. nodorum*. In a recent study, Wu et al. (2017) used a combined method between comparative genomics and GWA mapping by using 20 newly sequenced isolates of *Puccina triticina* from Australia. Based on 306,474 SNPs, the authors identified a polygenic architecture corresponding to 302 genes harboring at least one SNP associated with leaf rust virulence on wheat.

All the studies mentioned above were performed in controlled conditions or in common gardens with conditions that strongly differ from the habitats where the pathogenic microbes co-evolved with their natural hosts. Therefore, one of the next challenges is to perform phenotyping of the pathogenic trait of interest in more ecologically realistic conditions. In addition, because phytopathogenic microbes can be controlled by several members of the microbiota (Roux and Bergelson, 2016), it would be worthwhile to identify the genetic determinants in the pathogen species that are involved in the arms races with microbiota. Finally, as previously advised for plants, functional validation remains the gold standard to test whether the candidate genes identified by GWA mapping truly confer the aggressive/virulent phenotype to the microbial pathogen under study.

## A joint GWA mapping approach to draw a picture of the network of genetic interactions between plants and pathogens

Plant GWAS have been demonstrated to be successful in identifying genomic regions associated with disease resistance (Table 1), whereas microbial GWAS reporting genomic regions associated with pathogenicity are still in the starting blocks. Despite this increasing number of GWAS, it is interesting to note that GWA mapping has never been performed on the two counterparts of the plant pathosystem, either separately or jointly. Characterizing the molecular landscape of plant-pathogen interactions can considerably increase our knowledge on the co-evolutionary processes driving adaptive dynamics of plant species in plant communities (Allen et al., 2004; Karasov et al., 2014; Roux and Bergelson, 2016), thereby increasing our understanding and predictions on ED (Lambrechts, 2010; Roux et al., 2014a).

To study co-evolutionary quantitative genetics, the first step will require (i) estimating the respective contributions of plant and pathogen genetic variation to disease variation and (ii) estimating the heritability of plant × pathogen interactions ($H_{\text{GxG}}^{2}$) that can be calculated by dividing the phenotypic variance associated with “plant genotype—by—pathogen genotype” interactions by the total phenotypic variance across genotypes of both biotic partners (Roux and Bergelson, 2016). Because extensive phenotypic data sets are required for estimating the joint genetic effect of the plant and the pathogen, studies reporting heritability estimates of plant × pathogen interactions remains scarce in the scientific literature. This observation reinforces the need for the development of automated high-throughput phenotyping platforms.

**Figure 1:**
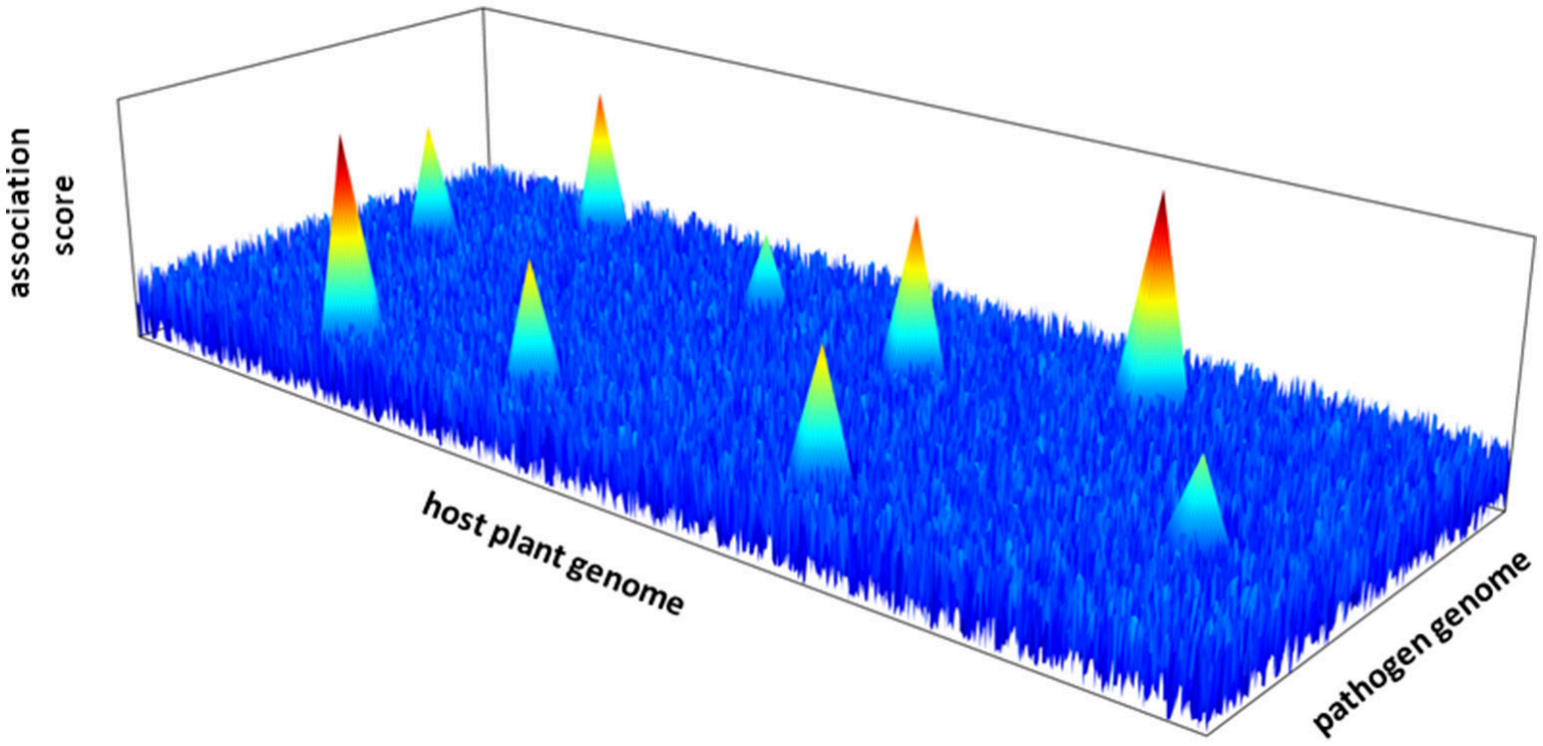
**Hypothetical 3D-Manhattan plot of joint GWA mapping between the genome of a host plant and a pathogen species**.

The next step requires the characterization of the genetic architecture of plant-pathogen interactions; that is the number of intergenomic epistatic QTLs, their physical locations in their respective genomes and their corresponding effect (Figure 1). Quantitative genetic methods have been developed to identify host-by-pathogen QTL interactions (Wang et al., 2006; Yang et al., 2008). These traditional QTL mapping based methods may however not be adapted to genome scans on both interacting species. New statistical methods must be developed with the challenge of performing joint GWA mapping by taking into account simultaneously the information provided by the genome sequences of the plant and the pathogen (Roux and Bergelson, 2016). An additional challenge relies on the correction for the effects of population structure of both interacting partners, by including separately the additive polygenic random effects of the plant species and the pathogen species.

While technically challenging, identifying in both biotic partners the genes (and more precisely the causative variants) that confer the quantitative phenotypic variants likely retained by natural selection will help to understand and predict co-evolutionary dynamics between a host and its pathogen either in natural populations or in crop fields.

## An ecological genomics approach to identify natural genetic variants driving the interactions between plants and microbial pathogens: a free-phenotyping joint GWA mapping approach

**Figure 2:**
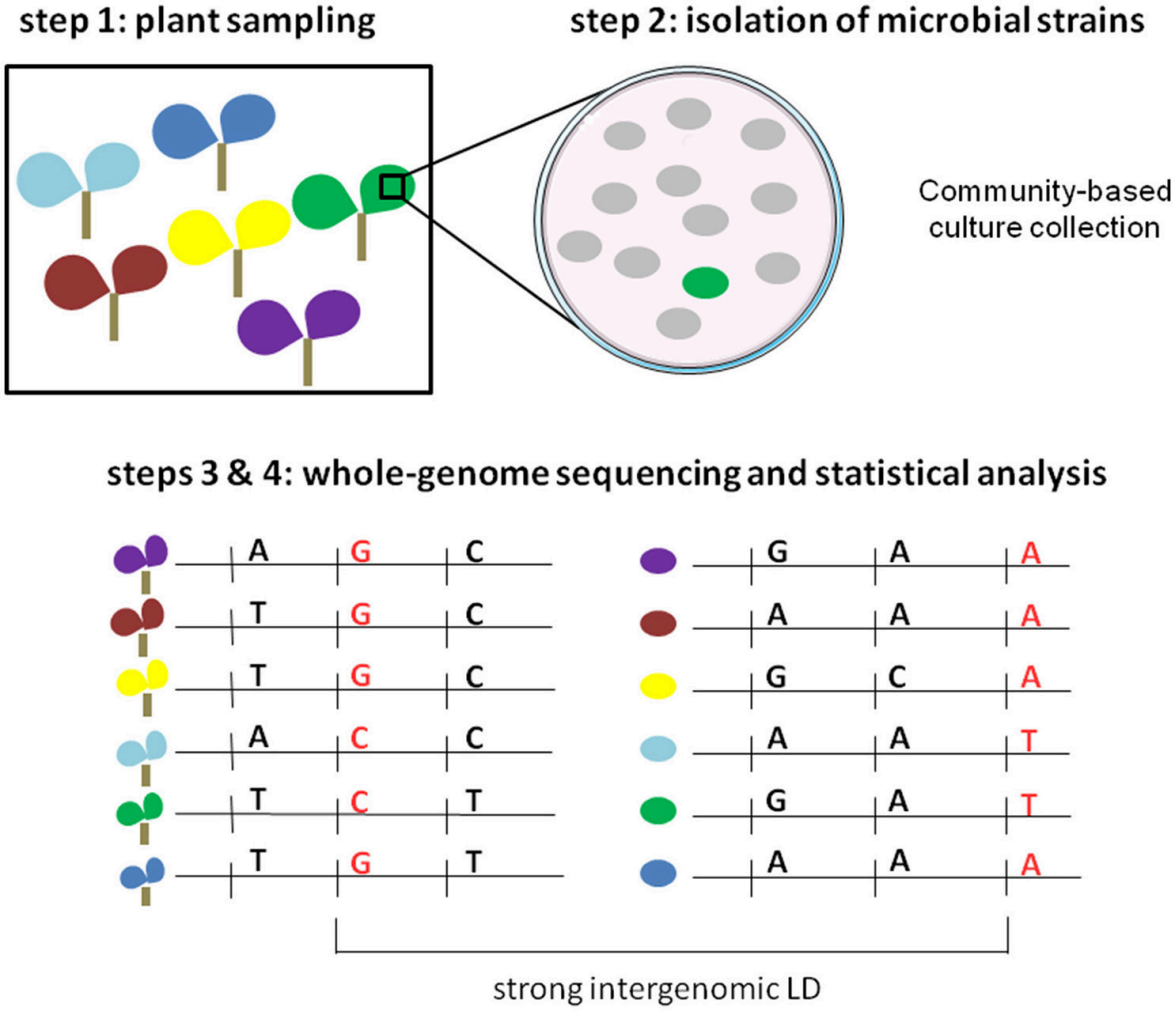
**Illustration of the four steps of the free-phenotyping joint GWA mapping approach**. Step 1: paired sampling of plants and microbiota in wild populations. Each color corresponds to a different plant population. Step 2: isolation of putative pathogenic strains. The green circle corresponds to the putative pathogenic strain whereas gray circles correspond to other members of the microbiota. Steps 3 and 4: whole-genome sequencing of both plants and microbial strains and genome-to-genome statistical analysis.

Based on coevolution signatures in host and pathogen genomes, an innovative free-phenotyping strategy was recently developed for global genome-to-genome analysis and employed in the human-HIV pathosystem (Bartha et al., 2013). Using paired human and viral data from 1,071 individuals, HIV-1 sequence variants were used as “phenotypic” traits to finely map human genetic variants in interaction with viral genetic variants. The authors demonstrated that using HIV-1 sequence variants was much more powerful than viral load to finely map human SNPs in the major histocompatibility complex (MHC) region that was already reported as implicated in human-HIV coevolution. By adopting an ecological genomics approach, this strategy can also be applied in plant pathosystems. Here, we propose four steps to detect highly significant associations between plant DNA polymorphisms and pathogen sequence variation, without the need to obtain large phenotypic data sets (Figure 2). Firstly, plants are sampled across a given geographic area in conjunction with the corresponding microbiota. If the demographic history of the plant species has been reported, the sampling should be limited to genetically homogeneous subgroups, allowing a reduction of false positives due to population structure during the genome-to-genome statistical analysis. Secondly, our method requires the isolation of one representative strain (putative representative pathogen strain) on each plant individual. Traditional microbiological methods for strain isolation and identification can be time- and material-consuming. However, the systematic identification of a conspicuous number of microbial strains can be strongly facilitated by combining community-based culture collections (CBC), housekeeping gene amplicon pooling (16S or *gyrB* for bacteria, 18S for fungi, ITS for fungi and oomycetes) and NGS technologies (Armanhi et al., 2016). With ever-cheaper genome-sequencing SMRT methods, the third step consists in generating paired plant and pathogen genomic data. The fourth step consists in performing joint association analysis using both host and pathogen genomes, with the lofty goal of identifying genetic polymorphisms in strong LD across the two genomes. Similarly to joint GWA mapping approaches based on phenotypic data, new statistical methods must be developed for testing gene-gene interaction while accounting for population structure including interactions between the genetic backgrounds of the two organisms.

Based on co-evolutionary processes, combining paired plant, and pathogen genomic information represents therefore an exciting opportunity, especially in wild species, to describe the molecular landscape underlying plant–pathogen interactions.

## Author contributions

FR supervised the project. CB and FR wrote the manuscript.

## Funding

This work was funded by the Région Midi-Pyrénées (CLIMARES project) and the LABEX TULIP (ANR-10-LABX-41, ANR-11-IDEX-0002-02).

### Conflict of interest statement

The authors declare that the research was conducted in the absence of any commercial or financial relationships that could be construed as a potential conflict of interest.
